# Quantifying Dynamic Balance in Young, Elderly and Parkinson's Individuals: A Systematic Review

**DOI:** 10.3389/fnagi.2018.00387

**Published:** 2018-11-22

**Authors:** Tarique Siragy, Julie Nantel

**Affiliations:** School of Human Kinetics, University of Ottawa, Ottawa, ON, Canada

**Keywords:** dynamic balance, Parkinson's disease, falls, walking stability, perturbations

## Abstract

**Introduction:** Falling is one of the primary concerns for people with Parkinson's Disease and occurs predominately during dynamic movements, such as walking. Several methods have been proposed to quantify dynamic balance and to assess fall risk. However, no consensus has been reached concerning which method is most appropriate for examining walking balance during unperturbed and perturbed conditions, particularly in Parkinson's Disease individuals. Therefore, this systematic review aimed to assess the current literature on quantifying dynamic balance in healthy young, elderly and Parkinson's individuals during unperturbed and perturbed walking.

**Methods:** The PubMed database was searched by title and abstract for publications quantifying dynamic balance during unperturbed and mechanically perturbed walking conditions in elderly adults and PD. Inclusion criteria required publications to be published in English, be available in full-text, and implement a dynamic balance quantification method. Exclusion criteria included clinical dynamic balance measures, non-mechanical perturbations, pathologies other than PD, and dual-tasking conditions. The initial database search yielded 280 articles, however, only 81 articles were included after title, abstract and full-text screening. Methodological quality and data were extracted from publications included in the final synthesis.

**Results:** The dynamic balance articles included 26 Coefficient of Variation of Spatiotemporal Variability, 10 Detrended Fluctuation Analysis, 20 Lyapunov Exponent, 7 Maximum Floquet Multipliers, 17 Extrapolated Center of Mass, 11 Harmonic Ratios, 4 Center of Mass-Center of Pressure Separation, 2 Gait Stability Ratio, 1 Entropy, 3 Spatiotemporal Variables, 2 Center of Gravity and Center of Pressure, and 2 Root Mean Square in the final synthesis. Assessment of methodological quality determined that 58 articles had a low methodological rating, a 22 moderate rating, and 1 having a high rating.

**Conclusion:** Careful consideration must be given when selecting a method to quantify dynamic balance because each method defines balance differently, reflects a unique aspect of neuromuscular stability mechanisms, and is dependent on the walking condition (unperturbed vs. perturbed). Therefore, each method provides distinct information into stability impairment in elderly and PD individuals.

## Introduction

### Parkinson's epidemiology

Parkinson's Disease (PD) is the second most common neurodegenerative disease in the world, with an increasing incidence within elderly individuals over the age of 60 (De Lau and Breteler, 2006; Hausdorff, 2009). It is well-established that people with PD are at an increased risk of falling due to gait instability with fall rates are as high as 70% (Hausdorff et al., 1998; Plotnik et al., 2007, 2008; Plotnik and Hausdorff, 2008; Hausdorff, 2009; Kerr et al., 2010; Nantel et al., 2011). Additionally, previous reports indicate that individuals with a fall history have an increased likelihood for subsequent falls thereby creating a self-perpetuating cycle (Hamacher et al., 2011; Francis et al., 2015). Therefore, accurately identifying individuals with unstable gait is crucial to determining fall risk as well as to assessing therapeutic intervention effectiveness for PD individuals.

### PD balance and gait issues

Parkinson's disease is a progressive neurodegenerative disease that causes loss of dopaminergic neurons that begins in the substantia nigra pars compacta propagating further into additional structures of the basal ganglia (Poewe et al., 2017). The basal ganglia is composed of several midbrain structures that rely on dopamine as a vital neurotransmitter to regulate movement (Blandini et al., 2000; Poewe et al., 2017). Dopamine reduction in PD causes impairment to the basal ganglia's function that results in PD's cardinal symptoms (bradykinesia, rigidity and tremor), which ultimately result in a multitude of motor impairments (Blandini et al., 2000; Poewe et al., 2017). Amongst these impairments is the disrupted gait pattern commonly observed in PD individuals. Indeed, previous research demonstrated that PD individuals ambulate with a reduced velocity, shorter stride lengths, increased double support time, reduced cadence and reduced interlimb coordination (Hausdorff et al., 2010).

Furthermore, parkinsonian gait is characterized by an increase in spatiotemporal variability that progressively worsens during the course of the disease (Hausdorff et al., 1998, 2010; Bloem et al., 2004). This has been attributed to the impaired basal ganglia in generating internal cues for rhythmic motor outputs (Hausdorff et al., 1998). Due its severity and implications for ensuing fall risk, increased spatiotemporal variability is considered a hallmark feature of Parkinsonian gait (Hausdorff et al., 2003; Plotnik and Hausdorff, 2008; Hausdorff, 2009). In addition, walking stability in PD is further threatened due to the paroxysmal phenomenon known as Freezing of Gait (FOG) (Nanhoe-Mahabier et al., 2011, 2013). Freezers, exhibit increased gait asymmetry and spatiotemporal variability, as well as reduced interlimb coordination when compared to non-freezers, even during optimally medicated states (Nieuwboer et al., 2001; Hausdorff et al., 2003; Bloem et al., 2004; Nantel et al., 2011).

### Quantifying dynamic balance

*Dynamic Balance* during steady-state gait is defined as the ability to stabilize an individual's COM within a series of alternating unilateral stances. Typically, clinicians utilize motor performance tests (e.g., Berg scale, Timed Up and Go, POMA, etc.) to assess dynamic balance and patient fall risk. However, previous research demonstrated several limitations in their ability to fully assess dynamic stability and predict fall likelihood in both healthy and clinical populations (Bhatt et al., 2011; Hubble et al., 2015). Therefore, several quantitative dynamic balance measures have been proposed as alternatives to assess fall risk (Hausdorff, 2009). However, despite these developments, ambiguity exists concerning which method is more suited to quantify dynamic balance for a particular demographic during both unperturbed and perturbed walking. This is primarily due to each test reflecting different properties of the neuromuscular system necessary for successful walking (Hausdorff, 2009).

With the widespan of dynamic balance measures, there is an apparent lack of uniformity in regards to which measure is most suitable for assessing dynamic stability, in both PD individuals and healthy adults, and in which specific scenario (perturbed or unperturbed walking; Hubble et al., 2015). This ambiguity is further perpetuated when one considers the various environmental conditions that may result in external perturbations.

### Quantifying dynamic balance recovery from mechanical perturbations

As mechanical perturbations (trips, slips, and surface conditions) can also disrupt an individual's balance, previous researchers suggested that dynamic balance measures must adequately evaluate the perturbations' destabilizing effects and their recovery therefrom. Current evidence on mechanical perturbations demonstrate that the neuromuscular system employs active recovery strategies to return the perturbed COM to a dynamic stability state (Marigold, 2002; Marigold and Misiaszek, 2009). Indeed, Marigold and Patla (2002) suggested that the neuromuscular system's ability to adapt to multiple balance conditions is rudimentary to maintain stability during walking (Marigold and Patla, 2002). However, during the course of natural aging, the ability to execute these recovery strategies in a timely manner becomes impaired. As PD is a neurodegenerative disease that predominately affects elderly individuals, this demographic is at increased risk to external perturbations debilitating effects. By quantifying an individual's dynamic stability level before and after perturbations, researchers can assess the effectiveness, or the lack thereof, of implemented recovery strategies. To accomplish this, it is necessary to determine the advantages and limitations of current quantitative dynamic stability methods in assessing balance and balance recovery from external mechanical perturbations.

### Systematic review purpose

Currently, there are a few literature reviews that examine and compare the various methods for quantifying dynamic stability (Hamacher et al., 2011; Bruijn et al., 2013). Existing literature reviews are limited in that they either only examine kinematic measures or do not conduct the review systematically. As such, the aims of this systematic literature review are (1) to examine the various methods of quantifying dynamic stability in PD individuals and healthy controls during steady-state walking and (2) to examine dynamic stability measures in recovering to a state of dynamic stability from external mechanical perturbations.

## Methods

### Identification of materials

This literature review was conducted in accordance with the PRISMA 2015 guidelines and protocol (Moher et al., 2009). The PubMed and Medline electronic databases were searched for publications assessing dynamic balance in healthy young, healthy older and people with Parkinson‘s Disease. The key search terms used included the following combinations:

1) “Gait balance” OR “locomotion balance” OR “ambulation balance” OR “walking balance” OR “dynamic balance” OR “falls” OR “falling”
    AND
2) “Walking perturbations” OR “walking trip” OR “walking slip”
    AND
3) “Adults” OR “Elderly Adults” OR “Parkinson's Disease”
    AND
4) “Faller” OR “Non-Faller”
    AND
5) “Freezers” OR “Non-Freezers”

Furthermore, reference lists of articles were scanned for any additionally relevant articles. If relevant articles were found, the PubMed and Medline databases were searched for full-text accessibility for inclusion in the article screening. If an article was not full-text accessible from the databases, the authors were not contacted. Articles searched were from 1990 to 2017.

### Inclusion/exclusion criteria

After removing duplicates, potential articles that were identified by the database search were independently screened by two researchers for relevancy. Discrepancies between screeners were resolved through discussion and comparison. Articles meeting the following inclusion criteria proceeded to data extraction:

1) Published in English in a peer-reviewed journal and was full-text accessible.
2) Unperturbed steady-state walking defined as consecutive strides after the Gait Initiation Phase and before Gait Termination.
3) Perturbed walking from mechanical perturbations defined as events that cause an external perturbing force to participants during walking. Methods comparing non-mechanical and mechanical perturbations were included.
4) The sample demographic was limited to either solely healthy young adults, elderly adults (fallers or non-fallers), and individuals with Parkinson's Disease (freezers or non-freezers) or was a comparison between any of the aforementioned demographics.
5) Implemented a quantitative measure of dynamic balance. Articles that examined both a clinical measure along with a quantitative measure were included.

Article exclusion criteria were as follows:

1) The sole use of a clinical gait assessment method (TUG, BBG, etc.) without an additional quantitative measure.
2) Pathologies outside of Parkinson's Disease. Articles also comparing Parkinson's Disease to another clinical population were excluded from the analysis.
3) All animal and modeling studies.
4) Sole use of non-mechanical perturbations defined as sensorimotor perturbations.
5) Backward and aquatic walking.
6) Literature reviews and meta-analyses.
7) Articles providing sensorimotor or instructional feedback.
8) Articles examining Gait Initiation and Termination. Current evidence demonstrates that each is a unique transitory phase between static balance and steady-state walking, and is governed by their own balance mechanics (Brenière and Do, 1991; Winter, 1995).
9) Protocols including sit-to-stand walking, lifting, bending, standing, chairs, stairs, running, inclined/declined treadmill, wheelchair, obstacle crossing, as well as gait initiation and termination.

### Data extraction and quality assessment

The same two researchers then independently extracted information from articles that passed both title and abstract screening as well as for methodological quality. Discrepancies were resolved through comparison and discussion. Extracted information from articles included: (1) author, (2) publication date, (3) dynamic balance quantification method, (4) study-design, (5) sample size, (6) sampling method, (7) demographic description, (8) inclusion/exclusion criteria, (9) unperturbed or perturbed walking, (10) type perturbation, (11) over-ground or treadmill walking, (12) number of strides, (13) walking duration, (14) Faller or Non-faller, (15) Freezer or non-Freezer, (15) Instrumentation.

To assess methodological quality, publications that proceeded to data extraction were assessed with a modified version of the Downs and Black Quality (Hubble et al., 2015). This checklist consists of 27-item that evaluates publications in regard to reporting, external validity, internal validity-bias, internal validity-selection bias, and power. General methodological quality was determined on 25 of the items with each having a corresponding single point. If publications met an item's criterion then one point was assigned to its score, if not then no point was assigned for that item. Additionally, no point was assigned if it was deemed unreasonable to determine if the item's criterion was met based on the publication's provided information. One item on the checklist assessed publications on a two-point scale based on reporting of principal cofounders. Specifically, two-points were given if all cofounders were listed, one-point if cofounders were only partially described, and zero points if none were described. The final item on the checklist assessed the publication's statistical power, which was measured on a five-point scale and carried more weight in the final total score. The higher the publications statistical power, the higher the score that was provided.

## Results

### Study selection

**Figure 1:**
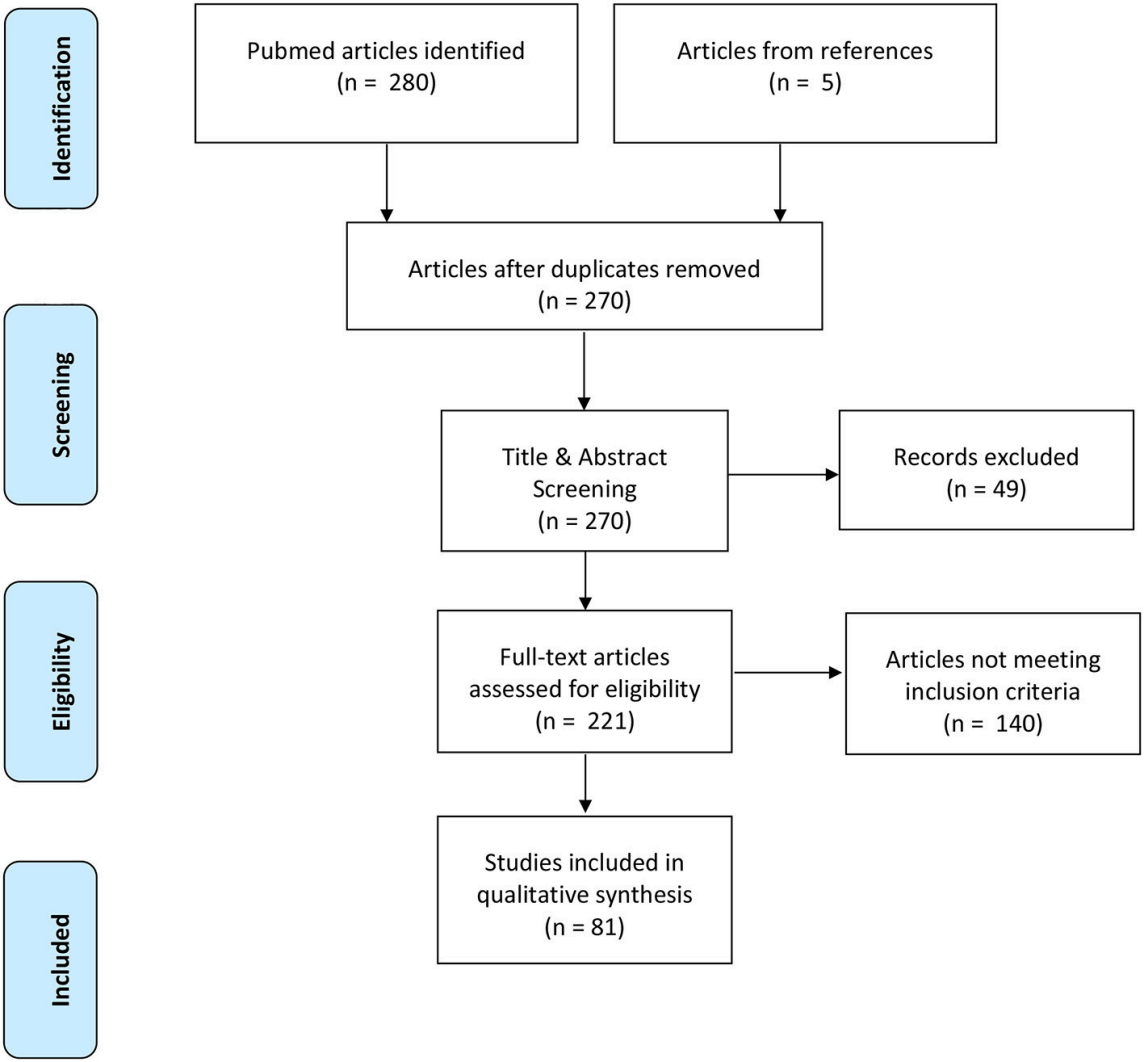
Prisma flow diagram highlighting the progression of the systematic review search and review processes.

The initial database search yielded 280 articles based on the defined search criteria (Figure 1) (Moher et al., 2009). Of the identified articles, 15 articles were removed as duplicates leaving 265 remaining publications for title and abstract screening. After title and abstract screening, 216 publications were deemed eligible for full-text screening based on their relevance to quantifying dynamic stability during walking. Full-text screening further excluded an additional 140 articles due to not meeting the predetermined inclusion criteria of this systematic review. The remaining 76 publications were included in the final synthesis and an additional 5 publications were included from manually scanning reference lists, thereby yielding a total of 81 publications for inclusion.

### Study characteristics

Several methods for quantifying dynamic stability during unperturbed and perturbed walking were identified with some articles quantifying balance with multiple measures: 26 Coefficient of Variation for Spatiotemporal Variability, 10 Detrended Fluctuation Analysis, 20 Lyapunov Exponent, 7 Maximum Floquet Multipliers, 16 Extrapolated Center of Mass, 11 Harmonic Ratios, 4 Center of Mass-Center of Pressure Separation, 2 Gait Stability Ratio, 1 Entropy, 3 Spatiotemporal Variables, 2 COG or COP, 2 Root Mean Square.

Of the unperturbed articles: 18 examined dynamic balance in healthy young adults, 30 examined elderly adults, and 15 examined PD individuals. The age range for young adults was from 22 to 35 years old, elderly adult age range was 60.6–84 years old, and age range for PD individuals was 60.2 to 71.9. The PD severity was commonly assessed by the Hoehn & Yahr (H&Y) scale and ranged between stages I-III. Additionally, scores on the Unified Parkinson's Disease Rating Scale (UPDRS) ranged from 13.8 to 36.1. Within the PD articles, two examined differences between ON and OFF. All studies reported details on age and generally provided an appropriate age-matched control group. Fourteen articles examined differences between fallers and non-fallers distributed between 12 articles in elderly adults and 2 in PD individuals. Definitions of fallers varied substantially between articles, however, only two articles examined falls prospectively. Two articles examined differences between freezers and non-freezers. In the perturbed walking articles 13 examined healthy young adults and 5 in elderly adults. No articles examined perturbed dynamic balance in PD individuals. The types and magnitude of the perturbations varied across studies. However, perturbed walking articles included 3 compliant surface (foam mat), 4 platform oscillations, 1 ML foot translation, 1 trunk pull, 5 slip, 2 trip, and 2 examined differences between both trips and slips. The articles were summarized into unperturbed and perturbed walking conditions then further divided by age group (young, elderly, and PD) and dynamic stability measure. All data extracted into the final synthesis are listed in Tables 1–3 for unperturbed walking in young, elderly and PD individuals, respectively and Tables 4, 5 for perturbed walking conditions in young and elderly individuals, respectively.

**Table 1:** Young adults unperturbed walking.

**Article**	**Experimental group**	**Quantification method**	**Number of strides**	**Overground or Treadmill**
Dingwell, 2006	Young adults = 10 (27.1 ± 3.2)	Maximum Floquet multipliers and Lyapunov exponent	Unstated	Overground
Bruijn et al., 2010b	Healthy adults = 9	Maximum Floquet multipliers and Lyapunov exponent	150	Treadmill
Hausdorff et al., 1995	Young adults = 10 (26)	Detrended fluctuation analysis	3,500	Overground
Dingwell and Cusumano, 2010	Young adults = 17	Detrended fluctuation analysis	213–334	Treadmill
Jordan et al., 2007	Young adults = 11 (24.9 ± 2.4)	Detrended fluctuation analysis, Coefficient of variation	Unstated	Treadmill
Terrier and Dériaz, 2011	Young adults = 20 (35 ± 7)	Detrended fluctuation analysis, Coefficient of variation, Lyapunov exponent	543	Overground and Treadmill
Stenum et al., 2014	Young adults = 10 (22.6 ± 2.8)	Lyapunov exponent	115	Treadmill
Dingwell and Marin, 2006	Young adults = 12 (26.7)	Lyapunov exponent	Unstated	Treadmill
Bruijn et al., 2009a	Young adults = 15 (23.6 ± 2.9)	Lyapunov exponent	50	Treadmill
Wu et al., 2016	Young adults = 24 (24.94 ± 1.43)	Lyapunov exponent	100	Treadmill
Dingwell et al., 2001	Young adults = 10 (27.1 ± 3.25)	Lyapunov exponent, Kinematic variability	30	Overground, Treadmill
Rosenblatt and Grabiner, 2010	Young adults = 10 (24.4 ± 4.5)	Extrapolated Center of Mass	Unstated	Overground, Treadmill
Yang and King, 2016	Young adults = 44 (23.9 ± 4.7)	Extrapolated Center of Mass	Unstated	Overground, Treadmill
Lu et al., 2017	Young adults = 12 (24.5 ± 2.3)	COM-COP separation	15	Overground
England and Granata, 2007	Healthy young adults = 19 (22.5 ± 2.8)	Lyapunov exponent	30	Treadmill
Ihlen et al., 2012a	Young adults = 10 (25 ± 4.7)	Lyapunov exponent	Unstated	Treadmill
Kang and Dingwell, 2006	Middle-age adults = 20 (40)	Lyapunov exponent	Unstated	Treadmill

**Table 2:** Elderly adults unperturbed walking.

**Articles**	**Experimental group**	**Faller or Non-faller**	**Quantification method**	**Number of strides**	**Overground vs. Treadmill**
Yack and Berger, 1993	Elderly adults = 20 (Fallers 78 ± 7, Non-fallers 77 ± 9.9) Young adults = 19 (24 ± 2.6)	Faller = any prior history of falling	Harmonic ratio	10	Overground
Auvinet et al., 2002	Young adults = 102 Middle-age adults = 100 Elderly adults = 82	n/a	Harmonic ratios	Unstated	Overground
Lowry et al., 2012	Very elderly adults = 13 (82.47 ± 2.2) Elderly adults = 13 (66.34 ± 2.6) Young adults = 13 (22.13 ± 0.9)	n/a	Harmonic ratios	Unstated	Overground
Brodie et al., 2015	Elderly fallers = 35 (79 ± 4) Elderly non-fallers = 61 (80 ± 4)	Fallers = >1 fall in the past year	Harmonic ratios	Unstated	Overground
Bisi and Stagni, 2016	Young adults = 10 (27 ± 1) Middle-age adults = 10 (45 ± 2) Elderly adults = 10 (67 ± 2) Very elderly adults = 10 (84 ± 2)	n/a	Harmonic ratios, Sample entropy	10	Overground
Granata and Lockhart, 2008	Young adults = 4 (26.3 ± 2.1) Elderly non-fallers = 4 (71.3 ± 6.5) Elderly fallers = 4 (71 ± 3)	Fallers = >2 falls in past 6 months	Maximum Floquet multipliers	35	Treadmill
Kang and Dingwell, 2009	Elderly adults = 18 (72.1 ± 6) Young adults = 17 (23.3 ± 2.6)	n/a	Maximum Floquet multipliers, Lyapunov exponent	Unstated	Treadmill
Malatesta et al., 2003	Young adults = 10 (24.6 ± 2.6) Elderly adults = 10 (65.3 ± 2.5) Very elderly adults = 81.6 ± 3.3)	n/a	Detrended fluctuation analysis, Coefficient of variation	20	Treadmill
Chien et al., 2015	Young adults = 10 (27 ± 4) Middle-age adults = 7 (50 ± 5) Elderly adults = 7 (70 ± 10)	n/a	Detrended fluctuation analysis, Coefficient of variation	200	Treadmill
Terrier and Reynard, 2015	20–29 years old = 20 (24.7 ± 2.8) 30–39 years old = 20 (34.6 ± 2.8) 40–49 years old = 20 (43.9 ± 2.9) 50–59 years old = 20 (54.8 ± 2.7) 60–69 years old = 20 (63.3 ± 3.2)	n/a	Lyapunov exponent, Walk ratio, Root mean Square	175	Treadmill
Toebes et al., 2012	Elderly fallers = 44 (63.3 ± 6.4) Elderly non-fallers = 90 (62 ± 6.1)	Fallers = >1 fall in the past year	Lyapunov exponent, Coefficient of variation	150	Treadmill
Ihlen et al., 2012b	Young adults = 10 (25.7 ± 4.7) Elderly adults = 10 (75.4 ± 4.6)	n/a	Lyapunov exponent, Coefficient of variation	Unstated	Treadmill
Toebes et al., 2015	Elderly adults = 134 (62.2 ± 6.2)	Fallers = >1 fall in the past year	Lyapunov exponent	150	Treadmill
Lugade et al., 2011	Young adults = 20 (23.6 ± 3.7) Elderly non-fallers = 10 (75.4 ± 7) Elderly fallers = 10 (78.9 ± 4.9)	One or more falls in the past year.	Extrapolated Center of Mass	Unstated	Overground
Mademli and Arampatzis, 2014	Young adults = 12 (25.2 ± 3.1) Elderly adults = 12 (68.2 ± 4.2)	n/a	Extrapolated Center of Mass	Unstated	Overground
Fujimoto and Chou, 2016	Young adults = 7 (22.1 ± 1.9) Elderly non-fallers = 15 (70 ± 3.2) Elderly fallers = 15 (71.9 ± 4.3)	Self-reported two or more falls in the past year.	Extrapolated Center of Mass	Unstated	Overground
Wright et al., 2015	Elderly non-fallers = 16 (72 ± 5) Elderly fallers = 24 (trip 71 ± 6, slip 68 ± 5)	Fallers = >1 fall in the past year	COM-COP separation	Unstated	Overground
Cromwell and Newton, 2004	Young adults = 20 (26 ± 3.4) Elderly adults = 17 (76.2 ± 6.9)	n/a	Gait stability ratio	Unstated	Overground
Hausdorff et al., 2001	Elderly adults = 52 (80.3 ± 5.9)	Fallers = >1 fall during 1 year follow-up	Coefficient of variation	Unstated	Overground
Helbostad and Moe-Nilssen, 2003	Elderly adults = 36 (72.5 ± 3.2)	n/a	Coefficient of variation	Unstated	Overground
Beauchet et al., 2009	Young adults = 30 (28.1 ± 6) Elderly adults = 33 (74.4 ± 7.1)	n/a	Coefficient of variation	6–12	Overground
Hurt et al., 2010	Young adults = 12 (24.5 ± 3.3) Elderly adults = 11 (60.6 ± 5.63)	n/a	Coefficient of variation	Unstated	Treadmill
Hausdorff et al., 1997	Young adults = 22 (24.6 ± 1.9) Elderly non-fallers = 17 (76.5 ± 4) Elderly fallers = 18 (82.2 ± 4.9)	Fallers = >1 fall in the past year	Coefficient of variation	Unstated	Overground
Brach et al., 2005	Elderly non-fallers = 81 (79.1 ± 3.9) Elderly fallers = 422 (80.3 ± 5.1)	Fallers = >1 fall in the past year	Coefficient of variation	Unstated	Overground
Brach et al., 2007	Elderly adults = 379 (79 ± 4.2)	n/a	Coefficient of variation	Unstated	Overground
Maki, 1997	Elderly non-fallers = 32 (81 ± 6.7) Elderly fallers = 43 (82.8 ± 6.2)	Prospective fallers who fell one or more times in the 1-year follow-up	Coefficient of variation, Classic spatiotemporal gait parameters	Unstated	Overground
Kavanagh et al., 2004	Young adults = 8 (23 ± 4) Elderly adults = 8 (74 ± 3)	n/a	Classic spatiotemporal gait parameters	10	Overground
Feltner et al., 1994	Elderly non-fallers = 11 (74.4 ± 1.7) Elderly fallers = 6 (71.7 ± 2.6)	Fallers = >1 falls in the past year	Classic spatiotemporal gait parameters	Unstated	Overground
Brach et al., 2010	Young adults = 30 (24.4 ± 4.3) Elderly adults = 30 (77.5 ± 5.1)	n/a	Harmonic ratios	Unstated	Overground
Kavanagh et al., 2005	Young adults = 8 (23 ± 4) Elderly adults = 8 (74 ± 3)	n/a	Harmonic ratios	Unstated	Overground
Mazzà et al., 2008	Young adults = 16 (24 ± 4) Elderly adults = 20 (72 ± 4)	n/a	Harmonic ratios	Unstated	Overground

**Table 3:** Parkinson's disease unperturbed walking.

**Articles**	**Experimental groups *N* (Mean age ± SD)**	**Disease severity**	**Quantification method**	**Number of strides**	**Overground or Treadmill**
Lowry et al., 2009	PD = 11 (68.92 ± 7.65) Elderly adults = 11 (68.92 ± 8.84)	***Hoehn & Yahr*** PD = 1.9 ± 0.8	Harmonic ratios	Unstated	Overground
Kirchner et al., 2014	PD = 19 (59.5 ± 10.2) Young adults = 20 (22 ± 2.7)	***Hoehn & Yahr*** I & II ***UPDRS*** 36.1 ± 13.5	Detrended fluctuation analysis, Coefficient of variation	25	Overground
Bartsch et al., 2007	PD = 29 (67 ± 1.3) *De novo* PD = 13 (68.9 ± 2.3) Elderly adults = 24 (64.3 ± 1.3)	Unstated	Detrended fluctuation analysis	Unstated	Overground
Frenkel-Toledo et al., 2005b	PD = 36 (61.2 ± 9) Elderly adults = 30 (57.7 ± 7)	***Hoehn & Yahr*** 2.1 ± 0.2 ***UPDRS*** 36.1 ± 11.5	Detrended fluctuation analysis, Coefficient of variation	Unstated	Treadmill
Cole et al., 2010	PD non-fallers = 17 (66.9 ± 2.1) PD fallers = 32 (66.2 ± 1.4) Elderly non-fallers = 17 (65.1 ± 2.1) Elderly fallers = 17 (70.2 ± 2.3)	***Hoehn & Yahr*** 1.8 ± 0.1 ***UPDRS*** 31.8 ± 2.3	Gait stability ratio, Coefficient of variation	Unstated	Overground
Plotnik et al., 2007	Young adults = 15 (26.3 ± 0.5) Elderly adults = 14 (69.1 ± 1.3) PD = 21 (71.9 ± 1.5)	***Hoehn & Yahr*** 2.3 ± 0.1 ***UPDRS*** 35.8 ± 2.6	Coefficient of variation	267	Overground
Nanhoe-Mahabier et al., 2011	Elderly adults = 15 (57.9 ± 7.3) PD freezers = 12 (60.5 ± 7.9) PD non-freezers = 15 (60.2 ± 9.2)	***PD freezers*** Hoehn & Yahr = 2.4 ± 0.3 UPDRS = 35.4 ± 8.9 NFOG-Q = 11.6 ± 5.3 ***PD non-freezers*** Hoehn & Yahr = 2.1 ± 0.3 UPDRS = 30.6 ± 7	Coefficient of variation	Unstated	Overground, Treadmill
Bryant et al., 2011	PD = 33 (70.61 ± 9.23)	***Hoehn & Yahr*** 2.58 ± 0.42 ***UPDRS “Off”*** 29.12 ± 11.36 ***UPDRS “On”*** 18.39 ± 8.55	Coefficient of variation	Unstated	Overground
Baltadjieva et al., 2006	Elderly adults = 22 (62.1 ± 11) *De Novo* PD Individuals = 35 (59.9 ± 13)	***Hoehn & Yahr*** 1.8 ± 0.5	Coefficient of variation	60	Overground
Blin et al., 1990	Elderly adults = 58 (72) PD = 21 (69.6)	***Hoehn & Yahr*** 2.8 ± 1.12	Coefficient of variation	Unstated	Overground
Barbe et al., 2014	PD non-freezers = 11 (63.5 ± 11.3) PD freezers = 11 (62.7 ± 12)	***PD non-freezers*** UPDRS “Off” = 31.7 ± 10.1 UPDRS “On” = 13.8 ± 7.2 ***PD freezers*** UPDRS “Off” = 29.45 ± 7.8 UPDRS “On” = 18.0 ± 5.8	Coefficient of variation	15	Overground
Herman et al., 2007	PD = 9 (70 ± 6.8)	***Hoehn & Yahr*** 1.5–3	Coefficient of variation	Unstated	Overground
Auriel et al., 2006	PD = 21 (70.2 ± 9.2)	***Hoehn & Yahr*** 2–3	Coefficient of variation	Unstated	Overground
Frenkel-Toledo et al., 2005a	Elderly adults = 30 (57.7 ± 7) PD = 36 (61.2 ± 9)	***Hoehn & Yahr*** 2.1 ± 0.2	Coefficient of variation	Unstated	Both
Latt et al., 2009	Elderly adults = 33 (67 ± 4) PD non-fallers = 33 (67 ± 4) PD fallers = 33 (67 ± 2)	***PD non-fallers*** Hoehn & Yahr 1 ***PD fallers*** Hoehn & Yahr 3	Harmonic ratios	Unstated	Overground

**Table 4:** Young adults perturbed walking articles.

**Article**	**Experimental groups *N* (Mean age ± SD)**	**Disease severity**	**Quantification method**	**Number of strides**	**Perturbation type**	**Overground or Treadmill**
MacLellan and Patla, 2006	Young adults = 8 (20.6 ± 1.7)	n/a	COM-COP separation	Unstated	Mat perturbations	Overground
McAndrew Young et al., 2012	Young adults = 12	n/a	Extrapolated Center of Mass	Unstated	Platform oscillations	Treadmill
Wang et al., 2012	Young adults = 16 (25 ± 3)	n/a	Extrapolated Center of Mass	Unstated	Trip	Overground
Yang et al., 2013	Young adults = 43 (26 ± 5)	n/a	Extrapolated Center of Mass	Unstated	Slip	Overground
Bhatt et al., 2013	Young adults = 32 (26 ± 4)	n/a	Extrapolated Center of Mass	Unstated	Slip and trip	Overground
Yang et al., 2014	Young adults = 36 (24.9 ± 3.7)	n/a	Extrapolated Center of Mass	Unstated	Slip	Overground
Rankin et al., 2014	Young adults = 10 (23 ± 1)	n/a	Extrapolated Center of Mass	250	ML foot translation	Treadmill
McAndrew et al., 2011	Young adults = 12	n/a	Maximum Floquet multipliers, Lyapunov exponent	150	Platform oscillations	Treadmill
Sinitksi et al., 2012	Young adults = 11 (26 ± 7)	n/a	Maximum Floquet multipliers, Lyapunov exponent	150	Platform oscillations	Treadmill
Chang et al., 2010	Young adults = 14 (25.2 ± 3)	n/a	Lyapunov exponent, Root mean square	100	Foam mats	Overground
Bruijn et al., 2010a	Young adults = 11 (27.7 ± 3.3)	n/a	Euclidean distances	140	Trunk pull	Treadmill
Ilmane et al., 2015	Young adults = 10 (22.3 ± 1.7)	n/a	Extrapolated center of Mass	Unstated	Slip and trip	Treadmill
McAndrew et al., 2010	Young adults = 12 (29 ± 7.5)	n/a	Coefficient of variation	Unstated	Platform oscillations	Treadmill

**Table 5:** Elderly perturbed walking articles.

**Article**	**Experimental groups *N* (Mean age ± SD)**	**Disease severity**	**Quantification method**	**Number of strides**	**Perturbation type**	**Overground or Treadmill**
Bhatt et al., 2011	Elderly adults = 119 (71 ± 6)	n/a	Extrapolated Center of Mass	Unstated	Slip	Overground
McCrum et al., 2016	Young adults = 11 (25.5 ± 2.1) Middle-aged adults = 11 (50.6 ± 6.4) Elderly adults = 14 (69 ± 4.7)	n/a	Extrapolated Center of Mass	Unstated	Trip	Treadmill
Yang and Pai, 2013	Elderly adults = 73 (72.6 ± 5.4)	n/a	Extrapolated Center of Mass	Unstated	Slip	Overground
Yang and Pai, 2014	Elderly adults = 187 (71.9 ± 5.1)	n/a	Extrapolated Center of Mass, Maximum Floquet multipliers, Lyapunov exponent, Classical gait parameters, Coefficient of variation	20	Slip	Overground
Menz et al., 2003	Elderly adults = 100 (79.9 ± 4)	Classified into fall risk based on tests of vision, peripheral sensation, strength, reaction time, and balance.	Harmonic ratios, Coefficient of variation	Unstated	Foam surface	Overground

### Methodological quality

**Figure 2:**
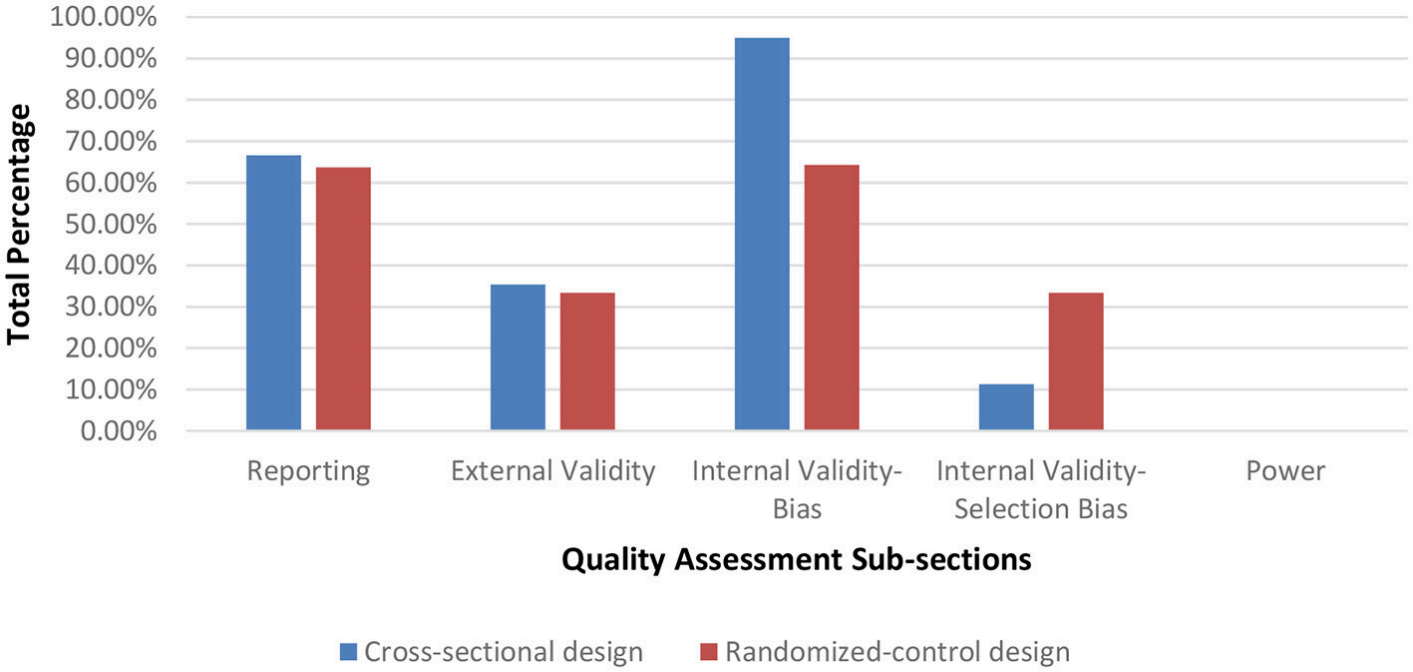
Average percentage scores on quality assessment sub-sections.

Seventy-nine of the assessed studies had a cross-sectional design while two studies used a randomized control protocol. After assessing methodological quality, 1 article was rated as having a very low methodological quality (range = 0–25%), 72 having a low methodological quality (range = 25.51–50%), and 7 having a moderate methodological quality (range = 50.1–75%). In general, the majority of the articles scored poorly on criteria for internal validity (selection-bias), external validity and on reporting the statistical power for their paradigms. Figure 2 displays the average percentage of each scoring for articles per category on the Downs and Blacks checklist sorted by study design. Articles scoring in the very low to low quality ranking are listed in Table A while moderately ranked articles are in Table B of the Supplementary Material.

## Discussion

The purpose of this literature review was to examine the various methods for quantifying dynamic stability during unperturbed and perturbed walking in elderly and PD individuals. As falls in these demographics occur primarily during walking, accurately assessing gait instability is crucial to determine those with increased fall risk. After full-text screening, 81 publications were assessed for methodological quality and included in the final article synthesis. From this total 63 publications examined dynamic stability during unperturbed walking (Table 1) conditions and 18 publications during perturbed walking conditions (Table 2). Of these articles, 1 article had a very low methodological quality, 72 articles had a low methodological quality and 7 articles had a moderate quality. Articles are grouped by methodological quality in Tables A, B in the Supplementary Material. The low score in the majority of the articles was due to many neglecting to report on threats to internal validity and *a priori* power Figure 2. Indeed, based on the information provided, we were unable to determine if the samples were representative of their respective population. According to Downs and Black, a poor rating on internal validity items introduces an inherent amount of bias into the study as these items were designed to assess if a study's sample truly represents its population, which is crucial as statistical measures are based on the assumption that an unbiased sample was drawn from the population. By reporting specifications on participant recruitment, researchers would ensure that they are minimizing the risk of unsubstantiated conclusions regarding their sample and the inferences drawn for the associated target population. Furthermore, none of the articles reported an *a priori* power analysis to determine if their sample size was sufficiently large to achieve statistical significance. Item 27 on the Downs and Black Checklist scores up to five points based on statistical power. Overall, the lack of reporting may be due to researchers being unaware of their necessity in assessments of methodological quality as both components are generally implied as criteria for quality research.

The following discussion will elaborate on the main quantification methods, as reported in the literature, in succession from unperturbed to perturbed conditions. Within each section, the method will be discussed in terms of the aspect of dynamic stability the respective method aims to quantify, how these measures are similar or different between demographics (young adults, older adults, and Parkinson Disease Individuals), the mechanisms of motor output associated with the method, the method's predictive ability in fall-risk assessment, and its limitations.

### Unperturbed walking methods

#### Spatiotemporal variability

Spatiotemporal variability is an overarching term that generally encompasses variability of stride time and length as well as step width. The variability in each of these parameters reflects a distinct aspect of motor control that contributes to stable walking. For instance, temporal parameters reflects the internal timing of the lower extremity (Hausdorff et al., 1998, 2003; Hausdorff, 2005). While spatial parameters, on the other hand, reflect an individual's ability to consistently spatially orient the lower extremity (Maki, 1997; Brach et al., 2005). However, spatial parameters can be further divided as current evidence indicates that separate motor processes control the lower extremity in the AP and ML directions (Kuo, 1999; Bauby and Kuo, 2000). Indeed, Bauby and Kuo (2000) demonstrated that lower extremity placement in the AP direction (stride and step length) is governed by automatic passive mechanisms while active mechanisms control ML placement (step width; Bauby and Kuo, 2000). Thus, each spatial parameter reflects a distinct aspect of motor control that contributes to stable walking. As the COM's trajectory dictates lower extremity placement, the neuromuscular system must integrate these multiple spatiotemporal parameters to successfully predict the COM's future dynamic state to avoid an uncontrolled fall (Brenière and Do, 1991; MacKinnon and Winter, 1993; Winter, 1995). Therefore, erratic spatiotemporal values reflect an inability to control extraneous COM movement within a rhythmic base of support. Previous research demonstrated that both aging and PD contribute to neuromuscular deterioration thereby threatening walking stability (Hausdorff et al., 1997, 2001; Brach et al., 2005; Baltadjieva et al., 2006; Nanhoe-Mahabier et al., 2011; Barbe et al., 2014; Kirchner et al., 2014). However, how aging and PD affects each spatiotemporal parameter specifically is inconsistently reported in the literature.

For instance, Malatesta et al. (2003) and Chien et al. (2015) reported increased stride time variability in healthy elderly adults compared to younger adults (Malatesta et al., 2003; Chien et al., 2015). In contrast, Hausdorff et al. (1997) demonstrated no differences in stride time variability between healthy elderly and young adults (Hausdorff et al., 1997). Additionally, aging's effect on spatial variability measures also yielded mixed results. When examining differences in step length variability between healthy elderly and young adults, Ihlen et al. (2012b) found increased variability in the elderly group (Ihlen et al., 2012b). The authors suggested that the increased variability reflects a reduced ability of elderly individuals to redirect their COM at the beginning of the double support phase (Ihlen et al., 2012b). Contrastingly, however, Beauchet et al. (2009) demonstrated that the increased stride length variability in their sample was due to elderly adults walking at a slower velocity and not due to aging effects (Beauchet et al., 2009). Instead, the authors suggested that aging directly accounts for increases in step width variability and thus is a more accurate characteristic parameter of elderly gait. Three additional studies demonstrated significant differences in step width variability between elderly and young adults. However, these differences were not consistently due to increased step width variability in elderly participants (Helbostad and Moe-Nilssen, 2003; Brach et al., 2005; Hurt et al., 2010). Indeed, Hurt et al. (2010) found reduced step width variability in elderly adults compared to their younger counterparts (Hurt et al., 2010). The authors explained the differences as elderly participants increasing voluntary control of their trunk in the ML direction thereby reducing stride width variability (Hurt et al., 2010). However, Brach et al. (2005) demonstrated that fall likelihood was closely associated with both low and high step width variability in elderly adults (Brach et al., 2005).

The disparity in the literature may be due to methodological issues in the samples collected. Indeed, the articles discussed thus far either did not account for participants' fall history or did so retrospectively. In two seminal articles, Maki (1997) and Hausdorff et al. (2001) examined spatiotemporal gait variability in elderly adults and their ability to prospectively identify fallers from non-fallers (Maki, 1997; Hausdorff et al., 2001). Maki (1997) demonstrated that fallers walked with increased stride length variability while Hausdorff et al. (2001) found increased stride time variability characterized elderly fallers (Maki, 1997; Hausdorff et al., 2001). A plausible explanation for the differences between fallers and non-fallers is that aging does not uniformly affect the motor control mechanisms responsible for rhythmic walking in all elderly adults thereby resulting in only some elderly falling (Hausdorff et al., 1997). It is possible that neurodegeneration in some elderly adults (fallers) is physiologically more akin to individuals with pathological conditions (Hausdorff et al., 1997). Indeed, four studies in this literature review reported increased spatiotemporal variability in PD individuals compared to healthy age matched controls. Three studies reported increased stride time variability and one study reported increased stride length variability in PD individuals compared to elderly adults (Blin et al., 1990; Frenkel-Toledo et al., 2005a; Plotnik et al., 2007; Nanhoe-Mahabier et al., 2011).

Gait is predominately considered an automatic motor process that is generated in subcortical structures with only limited supraspinal input to provide environmental information (Hausdorff, 2005, 2009). Increased stride time and length variability in PD and elderly fallers suggests that neurodegeneration, although stemming from separate pathological origins, affects the motor pathways involved in gait's passive and internal timing mechanisms (Hausdorff et al., 2001; Plotnik et al., 2007; Nanhoe-Mahabier et al., 2011; Kirchner et al., 2014). Additionally, based on the present evidence, aging seems to have a greater impact on the active mechanisms controlling walking stability in the ML direction as both fallers and non-fallers have altered step width variability compared to young adults (Brach et al., 2005; Beauchet et al., 2009; Hurt et al., 2010). However, there is currently a lack of literature that examined step width variability in PD individuals. As frontal plane walking stability is dependent on active sensory integration, an ability impaired in PD, examining step width variability would reflect ML foot placement strategies in this demographic (Bauby and Kuo, 2000). Overall though, it is interesting to note that some amount of variability is consistently reported across studies in healthy young and elderly subjects (stride time < 2%, stride length < 3%, stride width < 25%; Hausdorff et al., 1997; Maki, 1997; Brach et al., 2005). Brach et al. (2005) discussed the possibility that a moderate amount of variability is necessary for an individual to adapt to their environment (Brach et al., 2005). Thus, variability that is outside of this threshold may indicate both impairment for environmental adaptability as well as walking imbalance.

However, when quantifying spatiotemporal variability through the Coefficient of Variation, it is important to recognize this method's limitations. The Coefficient of Variation quantifies how much variability's magnitude contributes to the mean's value in any given gait parameter. While this method has the advantage of quantifying large spatiotemporal gait inconsistencies, it does not account for the variability's structure, which may provide additional information to the neuromuscular system's control of walking stability.

#### Detrended fluctuation analysis

In his seminal article, Hausdorff et al. (1995) demonstrated that variations in a gait time series are not random but exhibit long-range correlations, where one stride influences subsequent strides (Hausdorff et al., 1995). To quantify these correlations, Hausdorff et al. (1995) implemented the Detrended Fluctuation Analysis (DFA) method from Dynamical Systems Theory to quantify the amount of persistence (correlation) in a time series data (Hausdorff et al., 1995). When applied to gait, the DFA assesses the amount of correlation between stride intervals in a walking session along a spectrum, with lower values (approaching 0) indicating strides are uncorrelated and larger values (approaching 1) indicating greater stride correlation (Hausdorff et al., 1995; Frenkel-Toledo et al., 2005b; Jordan et al., 2007).

However, the degree to which stride intervals are correlated with one another in young, elderly and PD individuals varies considerably in the literature. Despite these discrepancies, an emerging pattern does appear demonstrating that long-range correlations naturally occur in healthy adults (Hausdorff et al., 1995; Jordan et al., 2007; Dingwell and Cusumano, 2010; Terrier and Dériaz, 2011; Chien et al., 2015). Indeed, six studies examined long-range correlations in healthy young adults and reported DFA values ranging from 0.72 to 0.81 (Hausdorff et al., 1995; Malatesta et al., 2003; Jordan et al., 2007; Dingwell and Cusumano, 2010; Terrier and Dériaz, 2011; Chien et al., 2015). However, it remains unclear as to how aging affects the presence of these long-range correlations. For instance, Malatesta et al. (2003) reported no differences in DFA values between young and elderly adults (Malatesta et al., 2003). Contrastingly, Chien et al. (2015) demonstrated that long-range correlations begin to deteriorate when adults reach middle-age (DFA values of 0.76 in young adults and 0.64 in middle-aged adults), although no further DFA break downs were demonstrated between middle-aged and elderly adults (Chien et al., 2015). As such, it remains unclear as to what effects aging have on the motor pathways responsible for stride interval correlations. Hausdorff et al. (1996) suggested that the presence of long-range correlations in healthy adults potentially reflect gait rhythmicity and automaticity generated by Central Pattern Generators (CPGs) in human subcortical structures (Hausdorff et al., 1996; Hausdorff, 2007, 2009). The contribution of CPGs to long-range correlations would explain the results found in PD. Three studies that investigated DFA in PD demonstrated reduced correlations in PD stride intervals compared to age-matched controls (Frenkel-Toledo et al., 2005b; Bartsch et al., 2007; Kirchner et al., 2014). For instance, Bartsch et al. (2007) reported DFA values of 0.72 in PD individuals and 0.80 in age-matched controls (Bartsch et al., 2007). Similarly, Kirchner et al. (2014) demonstrated lower DFA values in PD (0.76) compared to healthy elderly adults (0.93) (Kirchner et al., 2014). This suggests that each step, in PD individuals, is more independent and unrelated to previous steps indicative of gait fluidity impairment (Hausdorff, 2009). Therefore, previous research proposed that PD individuals continuously restart the motor process that controls stepping instead of building off of the lower extremity's previous stepping states (Hausdorff, 2009). Interestingly, however, Bartsch et al. (2007) reported no differences between *de novo* PD individuals and age-matched controls (Bartsch et al., 2007). Hausdorff (2009) suggested that deterioration in the long-range correlation motor pathways may not be an early symptom of the disease but stated that it remains unclear whether this is due to compensatory mechanisms or if damage to the basal ganglia is not yet severe enough to impact this parameter (Hausdorff, 2009).

Although the exact mechanisms responsible for long-range correlations remain inconclusive, their presence in human gait is well-substantiated. However, the studies included in this literature review also demonstrate that a certain amount of anti-correlation is present in healthy human gait (Hausdorff et al., 1996; Jordan et al., 2007; Dingwell and Cusumano, 2010; Terrier and Dériaz, 2011). Hausdorff (2009) proposed that this may be to reduce the risk of perturbations leading to “mode locking” or resonance and may reflect an individual's capacity to adapt environmentally (Hausdorff, 2009). Thus, suggesting that an optimal correlation threshold may naturally exist in healthy gait. In support of this theory, Hausdorff (2007, 2009) borrowed evidence from research examining heart beat signals that demonstrated when signals exceeded or receded from the correlation threshold observed in healthy individuals, it was indicative of cardiovascular disease (Hausdorff, 2007, 2009).

Overall, DFA results are currently difficult to interpret and there is limited research that demonstrates its predictive ability to quantify fall risk. Indeed, of all the studies included in this literature review, none of the authors examined differences between fallers and non-fallers. Therefore, it is difficult to draw a conclusion as to the DFA's ability to identify fallers or predict future fall risk. Furthermore, a comparison between results of different studies is difficult due to the lack of standardization in both the DFA's formulaic computation and the number of strides quantified, a value that directly affects the DFA's calculation.

#### Lyapunov exponent

Another common method that quantifies gait stability from a dynamical systems approach is the maximum Lyapunov Exponent. This method quantifies a system's average logarithmic rate of divergence after infinitesimal perturbations (Dingwell and Marin, 2006; Bruijn et al., 2009a, 2010b, 2013). The underlying notion of the maximum Lyapunov Exponent is that if a system's current state is altered from that of its previous state, then either state is deemed as perturbed from the other (Bruijn et al., 2013). When applied to human walking, these perturbations are considered to arise from “noise” in either the neuromuscular system or from the environment. Calculating the Lyapunov Exponent is dependent on the construction of a proper and comprehensive state-space that adequately defines the state of the system in any point in time (Dingwell et al., 2001). In the literature, the predominate method for state-space construction is derived from an anatomical segment's kinematic data, such as velocity, acceleration, position and jerk (Dingwell et al., 2001; Dingwell and Marin, 2006; Bruijn et al., 2009a, 2010a). When computed, the Lyapunov Exponent quantifies the rate of convergence (< 0) or divergence (>0) of the system's trajectory to its nearest neighboring trajectory in the reconstructed state space over the course of 0–1 strides (short-term Lyapunov Exponent) and 4–10 strides (long-term Lyapunov Exponent; Bruijn et al., 2009a). When the trajectories converge, the observed system is considered to have Local Dynamic stability while divergence indicates Local Dynamic Instability (Bruijn et al., 2009a, 2013). However, as only a link between the short-term Lyapunov Exponent and gait stability has been established, only this component will be discussed and referred to Bruijn et al. (2013).

Currently, results from studies using the Lyapunov Exponent are difficult to interpret due to several methodological discrepancies. For instance, it is unclear which anatomical segment is most appropriate for calculating Local Dynamic Stability (LDS) and the literature is currently divided between the trunk and lower extremity joints. Indeed, three studies calculated the Lyapunov Exponent on lower extremity joints while twelve studies based their calculations on trunk kinematic parameters (Dingwell et al., 2001; Dingwell and Marin, 2006; Kang and Dingwell, 2006, 2009; England and Granata, 2007; Bruijn et al., 2009a, 2010b; Terrier and Dériaz, 2011; Ihlen et al., 2012a,b; Toebes et al., 2012, 2015; Stenum et al., 2014; Terrier and Reynard, 2015; Wu et al., 2016). In a comparison between the lower and upper extremities, Kang and Dingwell (2009) demonstrated greater local dynamic instability in the lower extremity in healthy young adults (Kang and Dingwell, 2009). The authors explained their results as the greater LDS of the upper extremity, compared to the lower, is plausibly due to greater trunk inertia (Kang and Dingwell, 2009). Furthermore, the authors suggested that, due to the different anatomical properties of each extremity, different motor control mechanisms may be responsible for maintaining Local Dynamic Stability (Kang and Dingwell, 2009). As stable motion of each extremity is necessary for successful locomotion, comparison between them may not be feasible as they would reflect different aspects of neuromuscular control and dynamic balance.

However, two commonalities emerge in the literature regardless of the extremity assessed. First, it is apparent that a certain amount of local dynamic instability exists in healthy individuals. Of the publications that examined local dynamic stability in healthy young adults, all authors reported Lyapunov Exponents that were greater than zero thereby confirming divergence rates of neighboring trajectories in this demographic (Dingwell and Marin, 2006; England and Granata, 2007; Bruijn et al., 2009a, 2010a,b; Kang and Dingwell, 2009; Ihlen et al., 2012a; Stenum et al., 2014; Wu et al., 2016). This may be due to inherent biological noise of the neuromuscular system and may additionally reflect an individual's attempt to attenuate unintended trajectories to maintain dynamic balance. Secondly, the literature converges on the notion that this divergence increases due to aging thus demonstrating that elderly adults have reduced local dynamic stability compared to younger controls. Indeed, both Ihlen et al. (2012b) and Kang and Dingwell (2009) demonstrated that lower extremity local dynamic stability was significantly reduced in elderly compared to healthy young adults (Kang and Dingwell, 2009; Ihlen et al., 2012b). Ihlen et al. (2012b) suggested that this may indicate an inability of elderly adults in controlling their COM's direction (Ihlen et al., 2012b). Similarly, in regard to the upper extremity, the literature demonstrates that aging reduces the trunk's local dynamic stability (Kang and Dingwell, 2009; Terrier and Reynard, 2015). Terrier and Reynard (2015) examined Local Dynamic Stability between young, middle-aged, and elderly adults and demonstrated that the trunk's mediolateral (ML) dynamic stability decreased as a function of aging (Terrier and Reynard, 2015). Current evidence demonstrates that trunk ML stability is achieved by “active” motor control mechanisms to maintain the COM within the base of support's frontal plane boundaries (Bauby and Kuo, 2000). Therefore, the reduced ML trunk stability in elderly adults may indicate an impairment in this demographic's ability to active mechanisms to maintain frontal plane dynamic balance, which potentially may result in falling. Indeed, Toebes et al. (2012) demonstrated that trunk Local Dynamic Stability in the ML direction was effective at retrospectively identifying elderly fallers from non-fallers.

Additional evidence examining dynamic stability differences between fallers and non-fallers is currently limited, and it is unclear whether the reduced LDS in elderly fallers causes falls or develops as a result of already falling. Additionally, the current methods for state-space reconstruction, the number of strides examined, and the Lyapunov Exponent's formulaic computation are to-date unstandardized, making comparison between studies difficult to interpret. Finally, current evidence lacks a direct link that elaborates on the exact mechanisms of neuromuscular output involved in the control of dynamic stability and how these mechanisms are affected in the presence of PD. Indeed, during the course of this literature review, no studies were found that met the inclusion criteria that examined local dynamic stability in PD individuals.

#### Floquet multipliers

The last commonly employed method from dynamical systems theory is Maximum Floquet Multipliers (FM), which measures a system's orbital stability. This method quantifies how the current state of a system diverges or converges away from a nominal periodic cycle at a discrete point (Dingwell, 2006; Bruijn et al., 2013). When applied to gait data, the nominal period is calculated as the average gait cycle in a time normalized state space (reconstructed from trunk kinematic parameters; Dingwell, 2006; Granata and Lockhart, 2008; Kang and Dingwell, 2009; Bruijn et al., 2010b). Afterward, a gait cycle is compared to the nominal period (average gait cycle) at a fixed discrete point along a Poincare Section, to assess if the cycle converges or diverges away from the nominal (average) period. When Floquet Multipliers are below the value of one, the system is considered orbitally stable, however, if the value approaches or exceeds one, then the system is considered to be diverging from the nominal period thereby threatening its orbital stability (Dingwell, 2006).

Similar to the Lyapunov Exponent, the results from Floquet Multipliers are difficult to compare and contrast due to a lack of standardization in the reconstructed state-space, the number of strides investigated and in their formulaic computation. Therefore, conflicting evidence exists regarding its ability to determine stability and differentiate between different demographics. For instance, Granata and Lockhart (2008) found that elderly fallers had reduced orbital stability compared to elderly non-fallers and young adults (Granata and Lockhart, 2008). However, the authors reported no additional differences between healthy elderly and young adults (Granata and Lockhart, 2008). In contrast, however, Kang and Dingwell (2009) demonstrated that healthy elderly adults were less orbitally stable compared to healthy young adults (Kang and Dingwell, 2009).

The motor control implications from FM remain largely inconclusive. Nevertheless, based on the literature, it appears that healthy young and elderly adults are capable of preserving orbital stability by minimizing deviations from their nominal limit cycle. Specifically, of the evidence included, all publications reported FMs of less than one (Dingwell, 2006; Granata and Lockhart, 2008; Kang and Dingwell, 2009; Bruijn et al., 2010b). Previous research suggests that when values exceed the value of one, then orbital stability is considered lost, which leads to falling (Dingwell, 2006). Granata and Lockhart (2008) discussed the possibility that FM quantify the neuromuscular system's ability to return to the limit cycle by attenuating arising deviations, which if left unmodulated would continue to expand (Granata and Lockhart, 2008). In turn this would threaten an individual's orbital stability, thus theoretically increasing fall likelihood. However, it is unclear whether this ability deteriorates uniformly in an aging population or if certain individuals (elderly fallers) are more impaired. Furthermore, it is unclear whether this impairment is a consequence from prior falls or is a factor that leads to falling.

Additionally, several limitations to FM need to be considered when applying this method to gait research. It is important to note that FM are based exclusively on the assumption that the system being quantified is strictly periodic (Dingwell, 2006; Bruijn et al., 2010b). Biological systems, such as human walking, are considered stochastic in nature thus drawing into question FM's applicability to quantify gait stability (Ashkenazy et al., 2002; Bruijn et al., 2013). Furthermore, this method analyzes orbital stability at discrete time points to an average value and does not examine differences between neighboring trajectories (Bruijn et al., 2013). Finally, during the course of this literature review, no publications were found that examined orbital stability in PD individuals. Thus, in addition to the aforementioned limitations, further investigation of FM is necessary to determine its ability in identifying fall risk, uncover the motor control mechanisms that contribute to orbital stability, and whether PD affects these mechanisms.

#### Harmonic ratios

Based on harmonic theory, Harmonic Ratios (HR) quantify walking balance by examining the periodicity from an acceleration signal (Lowry et al., 2009, 2012). Since control of the COM is crucial for maintaining walking balance, harmonic ratios are typically applied to trunk acceleration data (MacKinnon and Winter, 1993; Winter, 1995; Auvinet et al., 2002; Lowry et al., 2012). When examining the anteroposterior and vertical trunk accelerations, harmonic ratios assume that continuous walking consists of regular stride patterns with each stride consisting of two steps (Auvinet et al., 2002; Lowry et al., 2009, 2012). Therefore, rhythmic and stable acceleration signals should repeat in even-numbered multiples to be considered “in-phase” with the stepping actions and result in a larger HR value (Auvinet et al., 2002). If, however, acceleration signals repeat as multiples of odd numbers then they are considered irregular and “out of phase” resulting in a smaller HR value (Auvinet et al., 2002). Overall, stability of an individual's walking pattern is then determined as a ratio of the summed amplitudes of the even to odd harmonics. In contrast to the AP and VT direction, rhythmic and stable acceleration signals in the mediolateral direction are characterized by multiples of odd numbers (Yack and Berger, 1993; Auvinet et al., 2002; Lowry et al., 2009, 2012). This is due to the fact that during heel strike, the COM is shifted in the frontal plane to the contralateral limb during stepping causing a monophasic, as opposed to biphasic, acceleration pattern during weight transfer in the double support phase (Auvinet et al., 2002).

Harmonic ratios, although grounded in a logical framework, is still an emerging method for quantifying walking stability. Of the publications found, conflicting evidence exists regarding HR's ability to differentiate trunk accelerations between demographics. For instance, Auvinet et al. (2002) and Lowry et al. (2012) demonstrated no differences between healthy young and elderly adults when participants walked at preferred walking speeds (Auvinet et al., 2002; Lowry et al., 2012). However, Bisi and Stagni (2016) reported a lower HR in the trunk's vertical accelerations for elderly adults over the age of 80 years old (Bisi and Stagni, 2016). Similarly, Yack and Berger (1993) found differences in the HR's of the anteroposterior and vertical accelerations in their “unstable” elderly participants compared to “stable” elderly and young adults (Yack and Berger, 1993). In opposition to the aforementioned studies, Kavanagh et al. (2005) demonstrated that elderly adults over the age of 70 years old exhibited lower HR's only in the trunk's mediolateral accelerations (Kavanagh et al., 2005). Furthermore, although still limited in the amount of research available, current evidence indicates a similar disparity in the trunk HR's for PD individuals. Indeed, two studies demonstrated lower trunk HRs for PD individuals compared to age-matched controls (Latt et al., 2009; Lowry et al., 2009). However, Lowry et al. (2009) reported lower HRs only in the mediolateral direction while PD participants in the study by Latt et al. (2009) had lower trunk HRs in all three movement planes (Latt et al., 2009; Lowry et al., 2009). Additionally, the authors reported differences between PD fallers and non-fallers in the anteroposterior and vertical planes (Latt et al., 2009).

Theoretically, rhythmic trunk accelerations indicate that the COM progresses between stance limbs along a smooth controlled trajectory (Auvinet et al., 2002). Control of the COM along the AP direction is considered largely “passive” as the COM's forward and downward momentum begins the stepping action with guiding input derived from the somatosensory system and subcortical cortical structures (Bauby and Kuo, 2000). Contrastingly, ML control is considered “active” as supraspinal input is required to determine lateral lower limb placement to stabilize the COM in this plane (Bauby and Kuo, 2000). Irregular COM acceleration may indicate an impairment in either the “passive” or “active” mechanisms responsible for COM movement. However, the disparity in the current literature makes it difficult to establish if and where impairments in trunk accelerations occur in elderly non-fallers, fallers and PD individuals. This disparity likely arises from the various methodologies used in each study. For instance, one issue arises from quantifying accelerations at different areas of the trunk. For instance, Yack and Berger (1993) as well as Mazzà et al. (2008) calculated HRs based on upper trunk accelerations (Yack and Berger, 1993; Mazzà et al., 2008). Contrastingly, Latt et al. (2009) and Brach et al. (2010) based their calculations on lower trunk accelerations (Latt et al., 2009; Brach et al., 2010). In a literature review, Winter (1995) collected evidence demonstrating that accelerations decreased in amplitude if measured on the upper trunk instead of the lower trunk (Winter, 1995). The author proposed that this is a stability mechanism whereby the neuromuscular system reduces perturbation amplitudes as they propagate toward the head in order to stabilize the visual field. As such, acceleration profiles between studies may not be comparable depending on whether the authors examined upper or lower trunk accelerations. A second cause for the lack of uniformity is caused by the different criteria used for participants. Auvinet et al. (2002) suggested that the inclusion criteria for participants, even within a specific demographic, will affect their gait performance (Auvinet et al., 2002). Future research should consider for example recording participants' activity level, fall history, and fear of falling level as these have been demonstrated to affect gait parameters and may potentially influence an individual's trunk acceleration patterns (Hausdorff et al., 1997; Toebes et al., 2012, 2015). Finally, it is important to consider that Harmonic Ratios do not directly quantify the COM in relation to the stability limits of the base of support (the lower extremity). As such, it is difficult to discern when altered trunk accelerations cause the COM to approach the base of support's stability limits.

#### Extrapolated center of mass

In classical biomechanics, walking stability is defined as maintaining the COM within a series of unilateral stances (Pai and Patton, 1997). Therefore, the COM's position in relation to the stability regions of the stance limb (range of the Center of Pressure) has been used as a method for measuring stability (Pai and Patton, 1997). However, Hof et al. (2005) suggested that quantification of COM-COP position alone is insufficient for assessing dynamic balance (Hof et al., 2005). The authors demonstrated that although the COM may lie outside the base of support, stability can be achieved if the COM velocity is directed toward the COP (Hof et al., 2005). Therefore, to accurately quantify dynamic balance, the Extrapolated Center of Mass (xCOM) was proposed as a single parameter that assesses both COM position and velocity in relation to the base of support (COP) (Hof et al., 2005). Stability is then determined by quantifying the distance between the xCOM and the base of support. Within the literature, a shorter xCOM-BOS distance indicates greater stability as the COM's dynamic state lies closer to the Base of Support's boundaries (Lugade et al., 2011; Süptitz et al., 2012; Mademli and Arampatzis, 2014; Fujimoto and Chou, 2016; Yang and King, 2016). Typically, the xCOM is quantified at heel-strike and toe-off as these points are considered more unstable due to the transfer of the COM between limbs over a smaller base of support (Lugade et al., 2011; Fujimoto and Chou, 2016; Yang and King, 2016).

When examining this method, a consistent pattern emerges when elderly fallers are compared to elderly non-fallers and healthy young adults. Indeed, both Lugade et al. (2011) as well as Fujimoto and Chou (2016) demonstrated that elderly fallers ambulate with a reduced xCOM-BOS distance compared to the other two age demographics in the sagittal plane (Lugade et al., 2011; Fujimoto and Chou, 2016). Less consistent differences were reported between elderly non-fallers and young adults (Lugade et al., 2011; Mademli and Arampatzis, 2014; Fujimoto and Chou, 2016). Indeed, Lugade et al. (2011) reported no differences between elderly non-fallers and young adults, however, both Fujimoto and Chou (2016) as well as Mademli and Arampatzis (2014) found that elderly adults walked with a reduced xCOM-BOS distance compared to younger controls (Lugade et al., 2011; Mademli and Arampatzis, 2014; Fujimoto and Chou, 2016). Additionally, Fujimoto and Chou (2016) demonstrated that when the xCOM was derived from the COM's acceleration data elderly non-fallers had even greater reductions in xCOM-BOS distances compared to young adults(Fujimoto and Chou, 2016). The authors suggested that acceleration may be more sensitive to age-related differences and that the altered acceleration profiles indicate an inability to control the COM's momentum to preserve balance (Fujimoto and Chou, 2016). Interestingly, out of all the included publications, only Lugade et al. (2011) examined mediolateral xCOM-BOS, despite instability in this plane being closely associated with increased fall risk, and reported no differences between elderly fallers, non-fallers and young adults (Lugade et al., 2011).

In general, findings in the AP direction indicate that elderly fallers employ a strategy to keep the xCOM closer to their base of support's boundaries(Lugade et al., 2011; Fujimoto and Chou, 2016). Current evidence demonstrates that elderly adults, particularly fallers, modify their gait, compared to young adults, by reducing their walking velocity and stride length, and increasing step width (Maki, 1997; Herman et al., 2005). The combination of these modifications has been termed as the “cautious gait strategy” (Maki, 1997; Herman et al., 2005). Yang and King (2016) proposed that individuals implement this strategy to control the COM's dynamic state in order to more readily return the COM within the Base of Support in a potential balance loss (Yang and King, 2016). However, “cautious gait” does not explain the lack of differences found in the mediolateral direction. Although increasing step width would increase an individual's lateral base of support, previous research demonstrates that this action simultaneously increases trunk acceleration and velocity (Rosenblatt and Grabiner, 2010). This in turn only maintains an individual's already existing frontal plane balance instead of enhancing it (Rosenblatt and Grabiner, 2010).

When quantifying walking stability with the xCOM several limitations should be considered. For instance, a paradox exists within the xCOM theoretical definition and what is reported in the literature. According to the evidence, an individual is considered more stable when the xCOM is closer to their BOS (Lugade et al., 2011; Süptitz et al., 2012; Mademli and Arampatzis, 2014; Fujimoto and Chou, 2016; Yang and King, 2016). However, based on the publications found, the demographic that displayed the closest xCOM-BOS distance were elderly fallers (Lugade et al., 2011; Fujimoto and Chou, 2016). In contrast, healthy young individuals consistently exhibited the largest xCOM-BOS distance compared to both elderly non-fallers and fallers (Lugade et al., 2011; Mademli and Arampatzis, 2014; Fujimoto and Chou, 2016). As such, the current definition of stability for the xCOM is counterintuitive as elderly individuals are established to have a substantially increased fall risk, particularly if a history of falling exists (Hausdorff et al., 1997, 2001). Lugade et al. (2011) proposed the possibility that an increased xCOM-BOS may indicate increased stability as an individual can handle more dynamical states of the COM (Lugade et al., 2011). In addition to this paradox, the xCOM is only capable of assessing COM velocity and position in relation to the base of support at discrete time points and is therefore incapable of determining temporal effects on these variables. Furthermore, this method is based on the inverted pendulum theory and does not account for the effects of segments that are not represented in this model (Hof et al., 2005). Lastly, there appears to be an inconsistency in how authors define the base of support in their studies. When Hof et al. (2005) proposed the xCOM the authors defined the BOS as the range of the COP (Hof et al., 2005). However, several publications neglected to report how they defined the BOS or provided alternatives to this method. A clear and standardized definition of the BOS would be beneficial when examining xCOM-BOS distance.

#### Unperturbed walking summary

Falling is one of the primary concerns for individuals with Parkinson's Disease and their caregivers. Therefore, there is a need for methods that can identify both individuals with fall risk and provide information on the neuromuscular system's impaired stability mechanisms. However, when examining the different quantification methods, it is important to consider how one defines stability. Indeed, this definition is crucial as it affects which anatomical structures are quantified (the lower or upper extremity), the quantification method itself, and the associated implications for motor control.

During walking, each extremity has a unique role that is controlled by different neuromuscular parameters. For instance, previous research proposed that CPG's control lower limb movement during walking (Hausdorff, 2009). Thus, lower extremity based methods may reflect the CPG's ability to control the stride-to-stride sequence. Evidence from spatiotemporal variability and detrended fluctuation analysis suggest that healthy adults are capable of stepping consistently (spatial variability), rhythmically (temporal variability) and in a correlated (DFA) manner culminating in regular stepping. In PD, however, these stepping processes are impaired to a degree beyond aging's effects. Indeed, compared to elderly adults, PD individuals walk with increased spatiotemporal variability and reduced long-range **correlations**. Plotnik and Hausdorff (2008) suggested that this increased variability is caused by impaired CPG output in this demographic (Plotnik and Hausdorff, 2008).

However, upper extremity stability may be controlled by alternative mechanisms. To accurately control the trunk during walking the neuromuscular system integrates multiple sensory systems to provide feedback information (Horak, 2006). As such, methods that quantify stability based on upper extremity parameters (Lyapunov Exponent, Floquet Multipliers, Harmonic Ratios) may reflect trunk stability mechanisms. The evidence from these various methods indicate that elderly adults are less capable of maintaining trunk stability compared to younger adults. Although still limited in research, these methods may hold direct implications for PD individuals due to their impairment in processing sensorimotor feedback.

Additionally, each quantification method reflects a unique aspect of neuromuscular control. For instance, both the Coefficient of Variation and DFA quantify variability in the lower extremity. However, the former quantifies variability magnitude while the latter quantifies variability in terms of its temporal structure. Hausdorff (2009) noted the magnitude and the temporal structure are two distinct characteristics and the value of each is independent of the other (Hausdorff, 2009). Similarly, Kang and Dingwell (2009) suggested that FM and the Lyapunov Exponent quantify different aspects of neuromuscular control as the upper extremity was more orbitally unstable and locally stable in both healthy young and older adults (Kang and Dingwell, 2009). Both methods quantify the divergence or convergence of variability in its own manner but are each based on specific assumptions about the investigated system. Finally, HRs and the xCOM both quantify the COM's kinematic state in relation to the base of support. However, HRs quantify stability through the trunk's cyclical movement, while the xCOM quantifies the trunk's motion state to the stability range of the base of support. As such, HRs represent an individual's ability to synchronize changes in trunk mechanics with that of the lower extremity. On the other hand, the xCOM indicates one's ability to withstand more dynamic conditions of the COM.

Overall, it appears that a certain amount of variability exists in the neuromuscular system and alterations outside this range increases fall likelihood. It is clear that aging and PD affect variability in some form indicating an altered capacity to process sensorimotor input, maintain rhythmical movement and execute corrective strategies. As walking is a complex skill requiring multiple neuromuscular control aspects and sensory input, implementing a single method to quantify stability is insufficient to reflect overarching balance impairment issues. Although all quantification methods have a grounded theoretical framework, additional research is necessary to determine their ability to predict fall risk in elderly and PD individuals. Indeed, only the Coefficient of Variation included studies that examined the differences between fallers and non-fallers prospectively thereby demonstrating its robustness in future fall prediction. Although methods, such as the Lyapunov Exponent, xCOM, and HR's examined differences between fallers and non-fallers, they did so retrospectively. Therefore, it is unclear whether these differences lead and caused the fall or if they arose only after fall onset. Thus, future research should consider prospectively examining potential differences within these demographics to determine each method's ability to predict falls. As elderly and PD individuals that have sustained a fall are at the greatest risk for future falls, quantifying dynamic stability with the Coefficient of Variation would assist clinicians in the early identification of individuals with unstable gait prior to fall onset. Additionally, implementing the Coefficient of Variation would assist clinicians in determining which aspect (passive or active mechanisms) are contributing to individuals' unstable gait. This subsequently would aid in the development of tailored gait therapy programs.

### Perturbed walking methods

#### Extrapolated center of mass

The xCOM was proposed as a single parameter that accounts for both the COM's position and velocity together (Hof et al., 2005). Previous research demonstrated that the Central Nervous System is capable of proactively and reactively adapting the COM's motion state (position and velocity) in relation to the BOS before and after perturbations (McAndrew Young et al., 2012; Wang et al., 2012; Yang and Pai, 2013). These adaptations are theorized to reflect an individual's feedforward (proactive) and feedback (reactive) mechanisms to maintain and return the COM's to a stable motion state (Wang et al., 2012; Yang and Pai, 2013).

Wang et al. (2012) demonstrated that in response to tripping, young adults reactively reduce their COM velocity while simultaneously shifting it posteriorly (Wang et al., 2012). This adaptive response would bring the perturbed COM's motion state closer toward the BOS and would help neutralize the trunk's forward angular momentum induced by the trip. Additionally, the authors demonstrated that after repeated trip exposure, young adults proactively reduced their COM velocity in anticipation of the upcoming trip (Wang et al., 2012). Similarly, Yang et al. (2014) demonstrated that young adults proactively and reactively shift their COM forward and reduced its velocity in response to induced slips (Yang et al., 2014). However, the aging process appears to have detrimental effects on an individual's ability to engage in adaptive responses. Indeed, McCrum et al. (2016) found a reduced rate of adaptation in elderly adults, compared to young and middle-aged, during the onset of initial perturbations (McCrum et al., 2016). The authors suggested that the reduced adaptation rate increases fall risk in this demographic when exposed to continuous perturbations, such as uneven walking surfaces. However, the authors further demonstrated that after multiple perturbation exposure, elderly adults exhibited the same adaptation magnitude as the young and middle-aged groups. This finding indicates that aging affects the feedback mechanisms responsible for perturbation onset recognition, which in turn delays their response in executing adaptation strategies (McCrum et al., 2016). In contrast, it appears that feedforward mechanisms remain largely intact over the course of aging as no differences were found between age groups after repeated perturbation exposure (McCrum et al., 2016). Current evidence suggests that reactive adaptations differ from proactive ones in that they are rapidly executed in response to afferent input (Yang et al., 2014). However, despite the unpredictability of perturbations outside a laboratory setting, previous research demonstrated that feedforward mechanisms can facilitate feedback-controlled recovery mechanisms (Yang et al., 2014; McCrum et al., 2016). Yang et al. (2014) demonstrated that in anticipation to upcoming slips, young adults proactively implemented a “cautious gait” strategy that causes their COM position to be shifted anteriorly and reduce their COM velocity (Yang et al., 2014). Furthermore, Yang and Pai (2013) demonstrated that elderly adults exhibited similar proactive adaptations after exposure to repetitive slips that reduced post-training the percentage of falls in their sample during a novel slip (Yang and Pai, 2013). It is well-established in the literature that this “cautious gait” strategy is a characteristic feature in elderly adults during unperturbed walking conditions (Maki, 1997; Herman et al., 2005). Thus, it is plausible that this may be an implemented strategy that attempts to facilitate feedback recovery mechanisms, through proactive adaptations, in the event of a potential environmental perturbation.

One limitation that must be considered when interpreting xCOM results from perturbation studies is the lack of uniformity as to how the COM's motion state is defined. Several researchers reported the COM's position and velocity in relation to the BOS separately as opposed to a single measure. We chose to include these authors in this section as both position and velocity, in relation to the BOS, are the two necessary components for the xCOM and still provide an adequate representation of an individual's balance recovery mechanisms. However, differences between individuals may be more distinct when both COM position and velocity are combined as indicated by previous work (Fujimoto and Chou, 2016).

#### Lyapunov exponent

Compared to unperturbed walking, relatively few articles were found that met this systematic review's inclusion criteria when examining the effect of perturbations on the Lyapunov Exponent's calculation of Local Dynamic Stability. Indeed, of the studies found, three examined the effect of minor surface perturbations and one examined an induced slip on participants' Local Dynamic Stability (Chang et al., 2010; McAndrew et al., 2011; Sinitksi et al., 2012; Yang and Pai, 2014). However, similar to unperturbed conditions, results from perturbation publications yield conflicting evidence.

For instance, McAndrew et al. (2011) reported that young adults had reduced Local Dynamic Stability (LDS), depicted by an increased Lyapunov Exponent, in all three cardinal planes (AP, ML, and VT) when perturbed by surface oscillations during walking (McAndrew et al., 2011). Additionally, the authors demonstrated that reductions in LDS were greatest when the Lyapunov Exponent was calculated in the same plane that the oscillation occurred in McAndrew et al. (2011). Similarly, Sinitksi et al. (2012) examined the effect between increasing amplitudes in ML surface oscillations and overground walking, and demonstrated that young adults were more unstable during perturbed trials than unperturbed but maintained LDS levels between oscillation amplitudes (Sinitksi et al., 2012). In contrast to these findings, no differences between surface conditions were found by Chang et al. (2010) when examining compliant foam surface and overground conditions (Chang et al., 2010). Furthermore, Yang and Pai (2014) demonstrated that LDS had a low ability in predicting falls from an induced slip in elderly adults (Yang and Pai, 2014).

Several methodological differences, in addition to the limitations mentioned previously, may account for the variable findings between studies. For instance, induced perturbations varied in type, intensity, and in their cardinal direction. In a literature review, Marigold and Misiaszek (2009) provided evidence that the neuromuscular system employs biomechanical recovery strategies that are specific to the encountered perturbation to maintain walking balance (Marigold and Misiaszek, 2009). Therefore, differences between Lyapunov Exponent values may reflect the various balance responses elicited by these specific perturbations. Additionally, these findings may indicate that certain walking conditions are more destabilizing to human walking than others. Indeed, Sinitksi et al. (2012) discussed the possibility that perturbation type has greater implications for walking stability than changes in perturbation amplitude (Sinitksi et al., 2012). However, previous research suggests that the Lyapunov Exponent has limited sensitivity in detecting local dynamic stability changes between various walking conditions due to the unstandardized methodology in its calculation (Bruijn et al., 2009b; Stenum et al., 2014). Furthermore, of the included publications, the Lyapunov Exponent was calculated based on trunk accelerometer data (Chang et al., 2010; McAndrew et al., 2011; Sinitksi et al., 2012; Yang and Pai, 2014). However, placement of the accelerometer varied between the Lumbar and Cervical regions amongst studies. As current evidence demonstrates that acceleration magnitudes diminish in superior trunk segments, this would affect the rate of divergence quantified by the Lyapunov Exponent thereby diminishing comparability between studies (Winter, 1995). Finally, the Lyapunov Exponent is only capable of quantifying a trajectory's divergence rate after infinitesimal perturbations (Bruijn et al., 2013). Thus, larger perturbations (trips or slips), may not be quantifiable with this method as they destabilize an individual globally (Bruijn et al., 2010a; Yang and Pai, 2014).

#### Floquet multipliers

Maximum Floquet Multipliers measures a system's convergence toward or divergence from a nominal (average) gait cycle due to neuromuscular noise or small environmental perturbations (Bruijn et al., 2013). Therefore, the majority of perturbation research on FM concentrates on the effect small surface perturbations, as opposed to trips or slips, has on an individual's orbital stability (McAndrew et al., 2011; Sinitksi et al., 2012; Yang and Pai, 2014). However, it is important to note that although a substantial amount of perturbation research was conducted on FM through modeling and robotics studies, relatively few experimental articles exist by comparison.

Of the studies included, two examined the effect small surface oscillations had on the trunk's orbital stability while one article examined FM's fall-predictive ability after an induced slip (McAndrew et al., 2011; Sinitksi et al., 2012; Yang and Pai, 2014). In a study of AP and ML surface oscillations on trunk orbital stability, McAndrew et al. demonstrated that heathy young adults became less orbitally stable in the direction of the induced oscillation (McAndrew et al., 2011). Additionally, the authors reported that participants were more sensitive to ML oscillations as the direction specific effects on trunk orbital stability were greatest in this direction (McAndrew et al., 2011). Indeed, Kuo (1999) demonstrated that humans are more unstable in the ML direction during walking (Kuo, 1999). Additionally, current literature has established that increased ML instability is closely linked with fall risk (Porter and Nantel, 2015). However, it is unclear how strong a destabilizing effect an individual can withstand before orbital stability is lost and a fall ensues. Sinitksi et al. (2012) reported that increasing surface oscillation amplitudes in the ML direction reduced orbital stability in the same direction in healthy young adults (Sinitksi et al., 2012). Additionally, the authors further stated decreases in orbital stability were marginal despite being statistically significant, therefore perturbation type may be more critical than magnitude (Sinitksi et al., 2012). This may explain findings by Yang and Pai (2014) who demonstrated that FM had a low predictive ability in differentiating fallers from non-fallers from an induced slip (Yang and Pai, 2014). However, an alternative explanation may account for these findings. As previously stated, FM are a measure of a systems convergence of divergence from a nominal period (average gait cycle; Bruijn et al., 2013). If FM quantify the neuromuscular system's capacity to modulate the continuous rise in deviations, as suggested by Granata and Lockhart (2008), it may be unable to detect large instantaneous stability threats, such as trips or slips (Granata and Lockhart, 2008). Overall, experimental perturbation research on FM are limited and further research is necessary to determine its suitability in quantifying perturbations effects.

#### Perturbed walking summary

Slips, trips, and uneven terrain pose a substantial threat to walking stability and account for more than a quarter of all falls in elderly adults. In response to perturbations, current evidence demonstrates that the neuromuscular system employs several proactive and reactive strategies to return the individual to a state of stability (Yang et al., 2014, 2016). However, the effectiveness of these strategies may deteriorate over the course of aging and in the presence of PD (McCrum et al., 2016). Therefore, determining appropriate methods that can quantify an individual's stability when encountering a perturbation are necessary to provide information into the capability of impaired neuromuscular functioning (aging and PD) in returning to a state of stability.

Previous research indicates that the neuromuscular system employs biomechanical recovery strategies that are specific to the encountered perturbation (Marigold and Misiaszek, 2009). As such, the types of perturbations, along with their associated recovery strategies, likely elicit different effects on dynamic stability measures and are likely non-comparable. Additionally, based on their theoretically framework, each quantification method may measure a distinct aspect of neuromuscular recovery. For instance, Floquet Multipliers and the Lyapunov Exponent measure convergence/divergence of a system's trajectory after infinitesimal perturbations and, therefore, may reflect smaller and more fine-tuned neuromuscular strategies to attenuate these miniscule deviations to a system‘s trajectory (Bruijn et al., 2013). Contrastingly, the xCOM may reflect larger strategies that preserve global stability through the aforementioned feedforward and feedback mechanisms (Yang et al., 2014).

Overall, it is clear that the perturbation type, quantification method utilized, and an individual‘s stability state prior to perturbation onset are critical factors when assessing perturbations‘ threat to walking balance. Interestingly, during the course of this literature review, a limited amount of research met our inclusion criteria when examining perturbations on PD individuals. Furthermore, no studies included examined differences in recovering dynamic stability in fallers compared to non-fallers. Previous research suggests that aging does not affect all individuals uniformly and fallers have altered motor control output that is similar to that of clinical populations (Hausdorff et al., 1997). Therefore, future research should consider examining recovery strategies in both fallers and PD individuals as their perturbation responses are plausibly altered compared to age matched controls. This is particularly important as both groups are continuously reported to have a high fall-risk (Bloem et al., 2004; Canning et al., 2014). Additionally, based on the included articles, it appears that methods stemming from Dynamical Systems Theory (Lyapunov Exponent and Floquet Multipliers) are limited in assessing the effects of perturbations. However, Bruijn et al. suggested that the stability state of the system (the individual) prior to a perturbation may affect the perturbation's destabilizing effects (Bruijn et al., 2010a). As such, future research should consider examining how much dynamical stability a system must exhibit to minimize destabilizing forces and whether this is altered in fallers and PD individuals. Furthermore, it is unclear how long it takes an individual (young, elderly, and PD) to return to a stability state post-perturbation. Thus, current clinicians should consider implementing the xCOM to quantify individuals' responses to perturbations. In doing so clinicians could not only determine potentially impaired feedback responses but also develop programs that facilitate the training of feedforward mechanism to reduce falls from perturbations.

## Limitations

Several limitations should be considered when interpreting the results of this review. For instance, we did not examine differences between overground and treadmill walking, which is demonstrated to affect several dynamic balance measurements. Additionally, we did not examine dual-tasking or feedback paradigms which provide valuable information into gait's cognitive and motor control processes. Furthermore, we did not examine differences between freezer and non-freezers or between ON and OFF medications both of which have been demonstrated to affect dynamic balance in this demographic. Additionally, findings from perturbed evidence in this literature should be considered with some limitations. First, relatively few articles that examined perturbation effects on Floquet Multipliers and the Lyapunov Exponent were found, due to the relatively new application of these methods to perturbation paradigms. As such, both methods warrant further investigation in the field for more in-depth conclusions. Secondly, we only examined mechanical perturbation literature and did not include sensory perturbations in our synthesis. Furthermore, limited research was present that examined mechanical perturbations' effects on fallers and PD individuals. As such, additional research is required to examine perturbation response strategies in both demographics. Additionally, as most of the articles had a low score on the Downs and Black checklist, this may have introduced unintended bias into our assessment when interpreting the results.

## Conclusion

After examination of the evidence, it appears that each quantification method provides unique information into dynamic stability control, or lack thereof, in young, elderly and PD adults. Therefore, several considerations should be given when selecting a quantification method as they each appear to reflect a unique aspect of neuromuscular control, which when impaired may contribute to falling in elderly and PD individuals. Considerations, such as the walking condition, perturbation type and magnitude, as well as if the upper or lower extremity should be quantified. Based on the evidence, future clinicians and researchers should consider the method of quantification carefully as to reflect the aspect of neuromuscular control that they wish to examine. Additionally, clinicians should consider using the Coefficient of Variation and the Extrapolated Center of Mass when, respectively, examining unperturbed and perturbed walking conditions in their clients. The articles examined indicate that the Coefficient of Variation is the most supported method in predicting future falls during unperturbed walking. While the Extrapolated Center of Mass provides a robust indication to the effectiveness of perturbation response strategies. As such, both methods can provide not only a robust quantitative assessment for fall risk but also insight into impairments to gait's motor control mechanisms.

## Author contributions

All authors listed have made a substantial, direct and intellectual contribution to the work, and approved it for publication.

### Conflict of interest statement

The authors declare that the research was conducted in the absence of any commercial or financial relationships that could be construed as a potential conflict of interest.
